# Real-world application of a scalable school-based physical activity intervention: A cross-sectional survey of the implementation of The Daily Mile in Greater London primary schools

**DOI:** 10.1371/journal.pone.0288500

**Published:** 2023-08-09

**Authors:** Bina Ram, Esther van Sluijs, Anna Chalkley, Dougal Hargreaves, Sonia Saxena

**Affiliations:** 1 Department of Primary Care and Public Health, Imperial College London, London, United Kingdom; 2 MRC Epidemiology Unit, School of Clinical Medicine, University of Cambridge, Cambridge, United Kingdom; 3 Centre for Physically Active Learning, Western Norway University of Applied Sciences, Bergen, Norway; 4 Centre for Applied Education Research, Wolfson Centre for Applied Health Research, Bradford Royal Infirmary, Bradford, West Yorkshire, United Kingdom; 5 Mohn Centre for Children’s Health and Wellbeing, Imperial College London, United Kingdom; University of the West of Scotland, UNITED KINGDOM

## Abstract

School-based physical activity interventions are considered ideal given their potential to reach most children. They can help children achieve the recommended guidelines of 60 minutes of moderate-to-vigorous physical activity per day. The Daily Mile is a popular school-based active mile intervention with a global reach. It recommends ten core principles for successful implementation, three of which are key for effectiveness: that it is quick (15 minutes), the whole school participates, and that it takes place in the school day during lessons (excluding physical education lessons and scheduled breaks). Studies assessing the impacts of The Daily Mile do not often report implementation of the ten core principles which is crucial to identifying the potential impact and feasibility of scalable interventions in real-world settings. Our aim was to assess adherence to The Daily Mile’s ten core principles in Greater London primary schools. We created and distributed a survey to 1717 primary schools during September 2020 and achieved a 21% (n = 369/1717) response rate by September 2021. Our sample was representative of Greater London primary schools with responses from every London borough. A total of 196/369 (53%) schools reported implementing The Daily Mile but none of them reported adherence to all ten core principles. Adherence to at least 6/10 principles in various combinations was reported by 54/196 (28%) schools. Only 19/196 (10%) schools that reported implementing The Daily Mile reported adherence to the three key principles recommended for effectiveness. Despite its popularity and global reach, our findings suggest that an implementation gap exists when The Daily Mile is adopted in real-world settings which is likely to challenge its intended purpose. Further research in school settings is needed to understand factors that can improve adherence to increase the potential public health impact of The Daily Mile and other similar interventions.

## Introduction

The World Health Organization (WHO) and many governments worldwide encourage school-wide physical activity promotion programmes as they have the potential to reach most children [1, 2]. These interventions can help children engage in regular physical activity during childhood and adolescence, which can help to improve physical fitness, cardiometabolic and bone health, and has also been shown to improve cognition and reduce symptoms of depression [2]. Physical activity during childhood has been shown to be a predictor of physical activity in adulthood [3]. However, in England, United Kingdom (UK), the number of children engaging in physical activity falls below the WHO’s recommendations of an average of 60 minutes of moderate to vigorous physical activity per day across the week [4]. According to UK Government official statistics, prior to the Covid-19 pandemic (2018 to 2019), 47% of children and young people (aged 5–16 years) self-reported to be engaging in 60 minutes of daily physical activity, but during the Covid-19 pandemic, this dropped to 45% [5]. Children’s physical activity measured objectively also showed a reduction compared with prior to the pandemic [6].

A growing evidence base suggests that school-based physical activity interventions are effective at reducing sedentary time and increasing time spent in physical activity [7, 8]. However, a systematic review in 2019 that included studies using objectively measured physical activity did not find school-based interventions to be effective in increasing children’s physical activity [9]. This may be due to less being known about how interventions are implemented or sustained in real-world settings. Most studies do not report an implementation assessment [10–12] thus creating a scientific evidence gap to support physical activity interventions in schools. Implementation assessments are crucial to understand the effectiveness of school-based physical activity intervention that may have the potential to change population health. Despite many school-wide interventions, success when delivered under real-world conditions or at scale is limited [13]. Research suggests that where there is deviation from implementation protocols, the intervention reduces impact [14]. There are disparities between the ideal of promoting physical activity interventions in school settings and current practices [15]. School-based physical activity interventions are often led by teachers who are motivated to implement the intervention, and usually without additional resource or support. Thus, implementation and sustainability may vary [15–17]. Furthermore, evidence suggests that implementation of school-based physical activity interventions are often adapted to fit around school or class requirements [18]. Whilst adaptations may occur for practical reasons, differences in implementation may dilute any beneficial impact on health outcomes [16, 17]. Previous research relating to school-wide physical activity interventions have called for a greater focus on documenting real-world implementation and sustainability [19].

In England, UK, the 2018 Childhood Obesity Plan outlined a national ambition for schools to include The Daily Mile, an active mile intervention, as an easy and accessible way to help increase children’s daily physical activity [20]. The Daily Mile recommends ten core principles for successful implementation which includes a whole school approach of children running or jogging for 15 minutes (‘a mile’), at least three times a week during the school day. Since its inception in 2012, The Daily Mile has had a global reach [21]. In England, 1 in 5 five state-funded primary schools are registered with The Daily Mile [22] and it appears to be reaching socio-economically disadvantaged areas and primary school populations which may need it most [23]. A growing evidence base of studies reporting effects of The Daily Mile intervention has demonstrated improved fitness in children [24, 25], but there is limited or inconsistent evidence of improved mental health [26, 27] or academic performance [28]. Most of these studies do not report an assessment of The Daily Mile implementation according to its ten core principles which may explain some of the limited or inconsistent findings of its effects [19, 24, 26, 28]. For example, while some have examined the implementation of The Daily Mile, they have not used a criterion-based assessment of implementation or adherence to the ten core principles; they reference some but not all principles which emerge anecdotally through qualitative methods of how teachers define and implement The Daily Mile [24, 29, 30]. Whilst these studies are important to understand the barriers and facilitators of implementing The Daily Mile in real world settings, they may be limited by subjective bias.

Given the calls for a greater focus on documenting real-world implementation and feasibility of physical activity interventions in schools [19], an assessment of The Daily Mile implementation is crucial to understand its potential public health impact. Therefore, the aim of this study was to establish adherence to The Daily Mile’s ten core principles in schools in one major conurbation.

## Materials and methods

### Study design

This is a cross-sectional quantitative study design. We approached all state-funded primary schools in Greater London, UK, to complete our survey. Characteristics of schools were derived from relevant publicly available data sources.

### The Daily Mile and its ten core principles

The Daily Mile is a teacher-led whole school active mile intervention. It involves children running or jogging for 15 minutes every day or at least three times a week during the school day [31]. It was founded by a former headteacher in Scotland in 2012 to improve the physical, social, emotional, and mental health and wellbeing of children [32] and has since been promoted by The Daily Mile Foundation.

There are ten core principles that The Daily Mile recommends for successful implementation (Table 1). Although The Daily Mile recognises that adaptations to its principles may be required to meet the requirements of the schools, they provide recommendations of how each principle should be implemented for success. In addition, The Daily Mile suggests that adherence to three of these principles are key for effectiveness. These are that it is quick (15 minutes), whole school participation, and that it is implemented at least three times a week during curricular lessons (i.e., not during scheduled breaks, prior to or after school, or during curricular physical education (PE) lessons).

**Table 1:** The Daily Mile’s ten core principles for successful implementation.

Principle		Definition
1.	Quick*	Just 15 minutes of running or jogging with no time spent changing, setting up or tidying up. Transitions between class and route should be slick.
2.	Fun	It should be fun for the children (not competitive). They can chat to their friends as they run along enjoying the experience together.
3.	100%*	It’s always fully inclusive–every child, every day. They should all be out together in the fresh air. Children with mobility difficulties should be supported to take part.
4.	Weather	Treat the weather as a benefit, not a barrier. Children enjoy being outside in the different types of weather, connecting with nature and being aware of the seasons.
5.	Route	Ideally, the route should have a firm and mud-free surface–most schools use the playground or an existing path. Incorporating child-pleasing loops and squiggles works well.
6.	Risk	Risk assess the route in order to ensure it is a safe activity.
7.	When to Go*	It should happen during curricular time, at least 3 times a week. Ideally, the class teacher should decide when to go out–they know their class and can respond flexibly to their needs.
8.	Clothes/shoes	The children run in their school clothes without changing into kit, putting jackets on if it’s cold or damp and taking sweatshirts off if it’s warm.
9.	Own Pace	The children go at their own pace. Done properly, it’s not a walk–able-bodied children should aim to run or jog for the full 15 minutes with only occasional stops to catch their breath, if necessary.
10.	Simple	Keep it simple. Resist the temptation to over complicate it. It should always be social and fun. From time-to-time, you may wish to connect it to the curriculum or do something seasonal, for example, running ‘Laps to Lapland.’

*Key principle recommended for effectiveness

(Source: The Daily Mile Foundation (https://thedailymile.co.uk/steps-to-success/)

### Survey design

A survey that had been previously created by London Marathon in conjunction with The Daily Mile, with support from Healthy Schools London and Mayor of London (S1 Fig), was used as the basis for our quantitative survey. Using the questions from the London Marathon survey, we met with our Steering Group Committee and Research Advisory Group regularly between November 2018 to March 2019 to develop additional questions that addressed each principle and captured adaptations. Questions and response options were piloted amongst our Steering Group Committee and Research Advisory Group separately. After the piloting process, we agreed on the wording of questions and response options, thus ensuring face validity of our survey. The final survey (S2 Fig) was approved by both our Steering Group Committee and Research Advisory Group in May 2019. We created the quantitative survey in two formats, paper-based (Microsoft Word 2010, Microsoft, Redmond, WA) and an online electronic version (Qualtrics XM). All responses were multiple choice with the exception of an optional comment box at the end of the survey.

### Public and patient involvement

Our survey was created in consultation with our Steering Group Committee and Research Advisory Group which includes representatives from The Daily Mile Foundation and sports organisations, public health professionals, health researchers, and former teachers.

### Participants and setting

We identified 1,747 Greater London state-funded primary schools up to August 2019 from a schools database created for a previous study [22]. By September 2020, 29 of these schools had closed, and two schools had merged, therefore our survey was distributed to 1,717 schools.

The invitation and survey were addressed to the headteacher of the school, but any teacher best placed to answer the questions was invited to complete the survey [33]. Access to the school name and corresponding responses were available to the lead author only during and after data collection.

### Procedure

During September 2020, the paper-based version of the survey was posted with a cover letter and a freepost return envelope to all the relevant schools identified in our database. A link to the online survey was also provided. Reminders to schools to complete the survey were adapted from procedures stated in our protocol due to the Covid-19 pandemic. We sent a reminder by email during November 2020, and a second email reminder in January 2021. A final reminder with a copy of the questionnaire was sent by post during April 2021.

### School characteristics

We accessed online data of ‘Schools, pupils and their characteristics’ [34] and ‘School performance’ tables [35] to obtain school characteristics of pupil numbers, pupil gender, number of pupils eligible for special educational needs (SEN) support, number of pupil’s with English and second language, number of pupils eligible for free school meals, and Ofsted rating (Ofsted is the UK’s ‘Office for Standards in Education, Children’s Services and Skills’) [36, 37] (S1 Table). School type was per the UK Government guidelines [38] (S1 Table).

As our study aimed to assess an intervention in primary schools (children aged 5 to 11 years), we used the Income Deprivation Affecting Children Index (IDACI) as a measure of the Index of Multiple Deprivation (IMD) [39] using a scale from 1 (most deprived) to 5 (least deprived).

### Data analysis

Responses from the postal surveys were entered into Excel 2010 (Microsoft, Redmond, WA); online responses were downloaded into Excel 2010 format. Of the surveys completed on paper, responses were recorded as ‘missing’ for any unanswered questions., There were no missing responses on the online version. Data were transferred into Stata v17 [40] and merged into one dataset. To identify whether there was adherence to The Daily Mile taking place during curricular lessons at least 3 times a week, we grouped together the responses to question 12 and question 20 (see S2 Table). Descriptive data are reported as *n* and percentages. We used the Chi-squared test to assess differences in school characteristics between those responding to the survey vs those that did not, and between schools implementing The Daily Mile vs those that were not. All descriptive analyses were conducted using Stata v17 [40]. Statements provided in the optional comment box at the end of the survey were grouped by reference to principles and participation and non-participation in The Daily Mile.

### Ethics

Ethical approval was granted by Imperial College London’s Research Ethics Committee (reference: 20IC6127). Completion of surveys was not compulsory. Consent was by survey completion; written consent was not required. Any identifying information (e.g., school name or teacher names) were removed for analysis. The protocol for this work has been published as part of a wider cohort study [33].

## Results

### Response rates

By September 2021, 369/1717 (21%) schools had completed the survey which included at least two schools from every Greater London borough. The surveys were completed by 170 class teachers, 112 headteachers/principals, 21 school administrators, and 64 other staff (e.g., school administrative staff). One staff member did not indicate their role.

### Participation in The Daily Mile

From the 369 schools that completed the survey, 196 (53%) schools reported that they were implementing The Daily Mile. Of these 196 schools, 124 (63%) were officially registered with The Daily Mile Foundation.

### School characteristics

School characteristics of those that did and did not respond to our survey were largely similar with the exception of the number or pupils eligible for free school meals, and area deprivation. Schools which responded to our survey were less likely to have pupils eligible for free school meals (20%), compared with pupils eligible for free school meals (23%) in schools that did not respond (S3 Table). Table 2 shows that there were no significant differences in school characteristics between schools that reported implementing or not implementing The Daily Mile.

**Table 2:** School characteristics of those reporting implementing and not implementing The Daily Mile.

Schools	Daily Mile n = 196	Non-Daily Mile n = 173	All n = 369	p value^b^
	n(%)^1^	n(%)^1^	n(%)^1^	
**Type of school** ^1^				
Academy converter	27 (14)	29 (17)	56 (15)	
Academy sponsor led	9 (5)	7 (4)	16 (4)	
Community	105 (54)	82 (47)	187 (51)	
Foundation	7 (4)	8 (5)	15 (4)	
Free	6 (3)	10 (6)	16 (4)	
Voluntary aided	42 (21)	36 (21)	78 (21)	
Voluntary controlled	0	1 (1)	1 (0.3)	0.64
**Total Pupil Numbers** ^2^				
<99	0	3 (2)	3 (0.8)	
100 to 499	154 (79)	138 (80)	292 (79)	
500 to 999	42 (21)	32 (19)	74 (20)	
> = 1000	0	0	0	0.15
**School pupil gender^1^**				
Mixed	195 (99)	172 (99)	1 (0.3)	
Boys	0	1 (1)	1 (0.3)	
Girls	1 (1)	0	367 (99)	0.37
**Pupil’s eligible for Special Education Needs (SEN) support^2^**				
< = 99	188 (96)	170 (98)	358 (97)	
100 to 199	8 (4)	3 (2)	11 (3)	
200 to 299	0	0	0	
> = 300	0	0	0	0.19
**Pupils with English as second language^2^**				
< = 99	47 (24)	48 (28)	95 (26)	
100 to 499	143 (73)	117 (68)	260 (70)	
500 to 1000	6 (3)	8 (5)	14 (4)	0.48
**Pupil’s eligible for free school meals^2^**				
< = 99	142 (72)	127 (73)	269 (73)	
100 to 199	52 (27)	40 (23)	92 (25)	
200 to 299	2 (1)	6 (3)	8 (2)	
300 to 399	0	0	0	0.23
**OFSTED rating^1^ ^3^**				
Outstanding	45 (23)	42 (24)	87 (24)	
Good	141 (72)	112 (65)	253 (67)	
Requires improvement	6 (3)	9 (5)	15 (4)	
Inadequate	0	0	0	
Rating not available	4 (2)	10 (6)	14 (4)	0.16
**Area deprivation IDACI⁴ (quintiles)**				
(Most deprived) 1	22 (11)	17 (13)	39 (11)	
2	44 (22)	49 (28)	93 (25)	
3	47 (24)	36 (21)	83 (22)	
4	52 (27)	48 (28)	100 (27)	
(Least deprived) 5	31 (16)	23 (10)	54 (15)	0.68

^a^May not total 100% due to rounding

^b^Chi-square test

^1^Source: https://www.gov.uk/government/statistics/schools-pupils-and-their-characteristics-january-2021

^2^Source: https://www.gov.uk/government/collections/statistics-performance-tables

^3^Ofsted rating of ’Inadequate’ includes schools that are rated ’Serious Weaknesses’ or ’Special Measures’

⁴IDACI: Income Deprivation Affecting Children Index

### Adherence to The Daily Mile’s ten core principles

Adherence to The Daily Mile’s ten core principles varied across the schools (Table 3). Most schools (87%) reported that The Daily Mile was implemented simply (i.e., no equipment was used), (86%) reported that it was implemented as a social and fun activity without a competitive element, 81% reported that no change of clothes/shoes was required, 77% reported that children ran or jogged at their own pace, and 67% of schools reported that The Daily Mile took place in the school playground as per the guidelines. Just over half of schools reported that The Daily Mile was quick (56%) and took place in all weathers (54%). However, under half of the schools that reported implementing The Daily Mile reported whole school participation (49%).

**Table 3:** Number of schools adhering and not adhering to the ten core principles of The Daily Mile.

Principle		n = 196 (%)
1	**Quick^a^** ^,^ *	
	At least 15 minutes^†^	110 (56)
	Less than 15 minutes	75 (38)
2	**Fun^b^**	
	Social and fun^†^	169 (86)
	Competitive element	16 (8)
3	**100%^c^ (whole school participation** *	
	Yes^†^	96 (49)
	No	90 (46)
4	**Weather (implemented in all weathers)^d^**	
	Yes^†^	106 (54)
	No	82 (42)
5 & 6	**Route & Risk^e^**	
	School playground^†^	131 (67)
	Other	17 (9)
7	**When to go^f^^,^** *	
	During lessons three times a week^†^	71 (36)
	Other^f^	117 (60)
8	**Change of clothes/shoes^d^**	
	Yes	30 (15)
	No^†^	158 (81)
9	**Run/jog at own pace^d^**	
	Yes^†^	150 (77)
	No	38 (19)
10	**Simple (no equipment used)** ^g^	
	Yes^†^	171 (87)
	No	16 (8)

^a^11 missing responses; At least 15 minutes includes responses of ‘15 to 30 mins’, ‘31 to 45 min’s and ‘46 mins or more’

^b^3 schools indicated implementing as a fun activity AND competitive element; 8 missing responses

^c^10 missing responses

^d^8 missing responses

^e^40 schools indicated school playground AND another area; other includes sports field, other sporting arena, local park, other; 8 missing responses

^f^Combined responses of two questions: when The Daily Mile takes place and how many times a week; other includes Daily Mile taking place once or twice a week and either during breaks between classes, lunch breaks, scheduled PE times, prior to the first lesson or after school; 1 school indicated during lessons AND outside of lessons; 7 missing responses

^g^9 missing responses

^†^Implemented as recommended by The Daily Mile

*Key principles recommended for effectiveness

Although 85% of schools reported that they implemented The Daily Mile during school lessons, and 65% reported that they implemented it at least three times a week, when combined, only 36% of schools had reported that The Daily Mile took place during school lessons and at least three times a week as per the principle recommends.

Overall, of the ten core principles, three were being adhered to by over 80% of schools (Principle 10 that it was simple, Principle 2 that it was fun, and Principle 8 that no change of shoes or clothes was required). The principles where under half of schools reported adherence to were Principle 3 (100%. i.e., whole school participation) and Principle 7 (taking place during lessons at least three times a week). These two principles are two of the three key principles recommended for effectiveness. Of the three key principles recommended for effectiveness (Principle 1 quick, Principle 3 100%. i.e., whole school participation, and Principle 7 taking place during lessons at least three times a week), only 19 (10%) schools reported implementing and adhering to all three as per the guidelines.

None of the 196 schools that reported implementing The Daily Mile adhered to all ten principles, at most six principles, in various combinations, were being implemented by 54 (28%) schools (Table 4).

**Table 4:** Total number of principles being implemented as per Daily Mile guidelines by number of schools.

Number of principles^a^	Schools, n = 196 (%)
0	8 (4)
1	0
2	0
3	3 (2)
4	11 (6)
5	31 (16)
6	54 (28)
7	48 (24)
8	34 (17)
9	7 (4)
10	0

^a^Total number of principles being implemented as per guidelines—number does not refer to which particular principles.

### Optional free-text comments

The comments provided additional information, some of which referenced a few of The Daily Mile principles especially where adaptations occurred, as well as reasons for participation or non-participation in The Daily Mile (S4 Table). For example, comments indicated that the children were bored of running or jogging therefore the introduction of a competitive element was needed to keep them engaged (Principle 2: quick), whilst others introduced a dance element or equipment when doing The Daily Mile (Principle 9: simple). Oher comments included that adopting The Daily Mile was dependent on the individual class teacher which limited whole school participation (Principle 3: 100%) or non-participation, and that it took longer than 15 minutes (Principle 1: quick) which was disruptive during lessons and therefore easier to implement during scheduled breaks or PE lessons (Principle 7: when to go). Some schools also commented that a change of shoes/clothes was required as not all children had suitable footwear (Principle 8: clothes/shoes). Reasons provided for non-participation in The Daily Mile commonly included lack of space and time constraints. However, schools implementing The Daily Mile provided positive experiences; that the children welcomed the break that The Daily Mile provided and enjoyed participation. Some teachers expressed that they noticed the effects of participation in The Daily Mile as the children were more focused and concentrated better.

## Discussion

Almost half of the primary schools that responded to our survey reported implementing The Daily Mile, but none of the schools reported adherence to all ten core principles. Adherence to the principles that were implemented varied widely across schools. Of the ten core principles, those which were most likely to be implemented as per the guidelines were that it was simple (Principle 10), fun (Principle 2), and that no change of shoes or clothes was required (Principle 8). This may suggest that these principles are more feasible to implement in real-world settings. Our findings also indicate that despite being identified as key, whole school participation (Principle 3) and that The Daily Mile should take place during lessons at least three times a week (Principle 7), appeared to be less feasible to implement and adhere to in practice. Only 1 in 10 schools reported adherence to all the three key principles which are crucial for equitable health benefits.

The Daily Mile was not implemented as a whole school approach and instead reported to be implemented to specific year groups. To improve population health, active mile interventions should be implemented as part of a whole school approach [41]. Whole school approaches to physical activity will ensure that they are embedded as part of sustainable measures to increase children’s physical activity. We also found that The Daily Mile was taking place during PE lessons or during scheduled breaks. Previous research has suggested that teachers count the time spent in The Daily Mile as part of the curricular two hours of quality PE lessons per week [29]. Such practices may reduce the benefits of participating in interventions intended as ‘additional’ activity to increase physical activity during the school day [42]. A process evaluation of The Daily Mile conducted in 2019 showed that children engaged in 13 minutes of moderate-to-vigorous physical activity (MVPA) during the intervention [43]. This was shown to be a significantly higher amount of time spent in MVPA compared with MVPA during PE lessons [44]. Other studies have also shown that The Daily Mile increases children’s MVPA between 5 to 15 minutes [25, 26] and improves fitness [25, 45–47]. However, these studies do not report when during the school day The Daily Mile takes place. If, as found in our study, where just over half of the schools reported implementing The Daily Mile outside of curricular lessons, children will not be receiving the ‘additional’ physical activity as intended by the intervention. A systematic review by Love et al., (2018) suggested that compensatory reductions in MVPA can occur in other parts of the school day when physical activity interventions are introduced at schools [9]. It is therefore difficult to establish from previous studies whether the MVPA during The Daily Mile would have otherwise been accumulated elsewhere in the school day. Our findings add important contributions to the feasibility of scalable interventions in the real-world by illustrating the challenges for development and implementation of public health interventions in school settings.

Some studies that have assessed the impacts of The Daily Mile on children’s health have captured why adaptations to implementation occur. For example, a mixed-methods study by Routen et al., (2021) explored how The Daily Mile works in practice and included questions relating to The Daily Mile’s ten core principles (including the three key principles for effectiveness) as part of their semi-structured interviews [48]. They found that implementing the principles as per the guidelines were difficult to deliver in practice; teachers stated that it took longer than 15 minutes to allow children to change into appropriate footwear or outerwear depending on weather, whilst being mindful that pupils from disadvantaged backgrounds may not own suitable footwear for all weather conditions [48]. They also found that teachers reported creating their own version of The Daily Mile to work in the space available to them and to keep it interesting for the children [48]. In our study, comments in our optional comment box where adaptations to the principles were referenced supports the research by Routen et al. (2021). It is important to note that our results reflect teacher responses during the Covid-19 pandemic where schools were adhering to restrictions. Although some teachers commented that they had to stop implementing The Daily Mile, they also noted how they implemented The Daily Mile prior to the restrictions. Therefore, the adaptations to implementation that we have captured in our survey supports that of other qualitative studies which have identified barriers and facilitators of The Daily Mile in practice [48–50].

As shown in our study, differences in the implementation of The Daily Mile across schools suggest that real-world evidence is needed to identify adherence to the underlying features of The Daily Mile. Our findings indicate deviation from the protocol of how to effectively implement The Daily Mile (adherence to the 10 principles) therefore it can be assumed that there is a decrease in benefit when grass roots interventions like The Daily Mile are scaled-up as shown in population measures of children’s physical activity (i.e., the Active Lives Survey).The Daily Mile’s ten core principles were developed based on practitioner experience of what would be effective. The principles are advantageous in terms of school and teacher acceptability of a grass-roots development, but with the absence of behaviour change theory which is crucial to plan, deliver, and evaluate health promotion interventions successfully [51], it is challenging for researchers to examine The Daily Mile’s effectiveness or of other similar interventions. Without an assessment of implementation, there are difficulties to conclude whether the lack of evidence to support physical activity interventions in schools are due to program failure or implementation failure [52]. This is likely to have implications for policy research and practice.

### Strengths and limitations

Strengths of our study include that it is the first study to quantitatively assess the implementation of The Daily Mile based on their criterion for successful implementation, in a real-world setting. The large-scale assessment across a breadth of schools in a diverse major urban conurbation to provide a wider perspective of a scalable intervention is an important strength of our study. Previous qualitative studies have assessed implementation between 4 and 8 schools [24, 29, 30]. Using the ten core principles of The Daily Mile as a criterion based assessment of implementation allows for study replication, and highlights the importance of including an intervention implementation assessment when assessing health impacts. Our findings may be representative of the wider-school population in a major conurbation which is socio-economically varied and ethnically diverse as we achieved survey completion from at least. two schools from every London borough one of which reported implementing The Daily Mile. However, small but significant differences found in two school characteristics (number or pupils eligible for free school meals and area deprivation) between the schools that reported implementing The Daily Mile compared with those that did not, may limit the generalisability of our findings. There are also important limitations to our work. Although our survey included face validity, we were unable to measure the concurrent validity or reliability of our survey as we did not have accessible information on the validity or reliability of the London Marathon survey. Therefore, our findings should be interpreted with caution. Furthermore our survey was not piloted due to being undertaken during Covid-19 restrictions. However, as the survey was co-designed with teachers and wider school-stakeholders, we ensured that the questions were appropriate, and the wording was comprehensible. The surveys were completed at a school level which would increase the reach of the survey, however completion at a class level may have provided more accurate responses. Our low response rates may have been due to several reasons. For example, we distributed the survey during the Covid-19 pandemic, although schools had re-opened, there were strict measures in place. In addition, whether our survey was forwarded on to the person best placed to complete the survey was beyond our control. Our low response rates therefore may limit generalisability. Some of the schools had provided comments that they had stopped implementing The Daily Mile due to school pandemic restrictions and their responses reflected a non-Daily Mile school. In addition, we were unable to distinguish the number of schools that implemented The Daily Mile for 15 minutes as per the recommendation. This was due to the response option of ‘15 minutes to 30 minutes’ being provided. The Daily Mile taking more than 15 minutes may not necessarily be perceived as ‘quick’.

## Conclusions

An implementation gap exists when The Daily Mile is adopted by schools which may impact the intended effect. We recommend the inclusion of implementation assessments when examining the impact of The Daily Mile and other similar interventions. This would strengthen the evidence base for identifying school-based physical activity interventions that are feasible to adopt in real-world settings and help to determine sustainability of these interventions in the longer-term thus informing future school-based physical activity policies.

## Supporting information

S1 Checklist STROBE 2007 (v4) statement—Checklist of items that should be included in reports of *cross-sectional studies*. (DOCX)

S1 Fig London Marathon survey. (TIF)

S2 Fig School survey. (ZIP)

S1 Table Ofsted ratings and school type descriptions. (PDF)

S2 Table Grouping survey responses. (PDF)

S3 Table School characteristics by survey completion. (PDF)

S4 Table Optional free-text comments. (PDF)
