# Correction: Real-world application of a scalable school-based physical activity intervention: A cross-sectional survey of the implementation of The Daily Mile in Greater London primary schools

**DOI:** 10.1371/journal.pone.0328373

**Published:** 2025-07-15

**Authors:** Bina Ram, Esther van Sluijs, Anna Chalkley, Dougal Hargreaves, Sonia Saxena

The following information is missing from the Acknowledgements section: The authors thank The Daily Mile Foundation for funding and support.
