# Supplementary figures and images for: Real-world application of a scalable school-based physical activity intervention: A cross-sectional survey of the implementation of The Daily Mile in Greater London primary schools
Source: PLoS One. 2023 Aug 9;18(8):e0288500. doi: 10.1371/journal.pone.0288500 (PMC10411754; doi:10.1371/journal.pone.0288500)

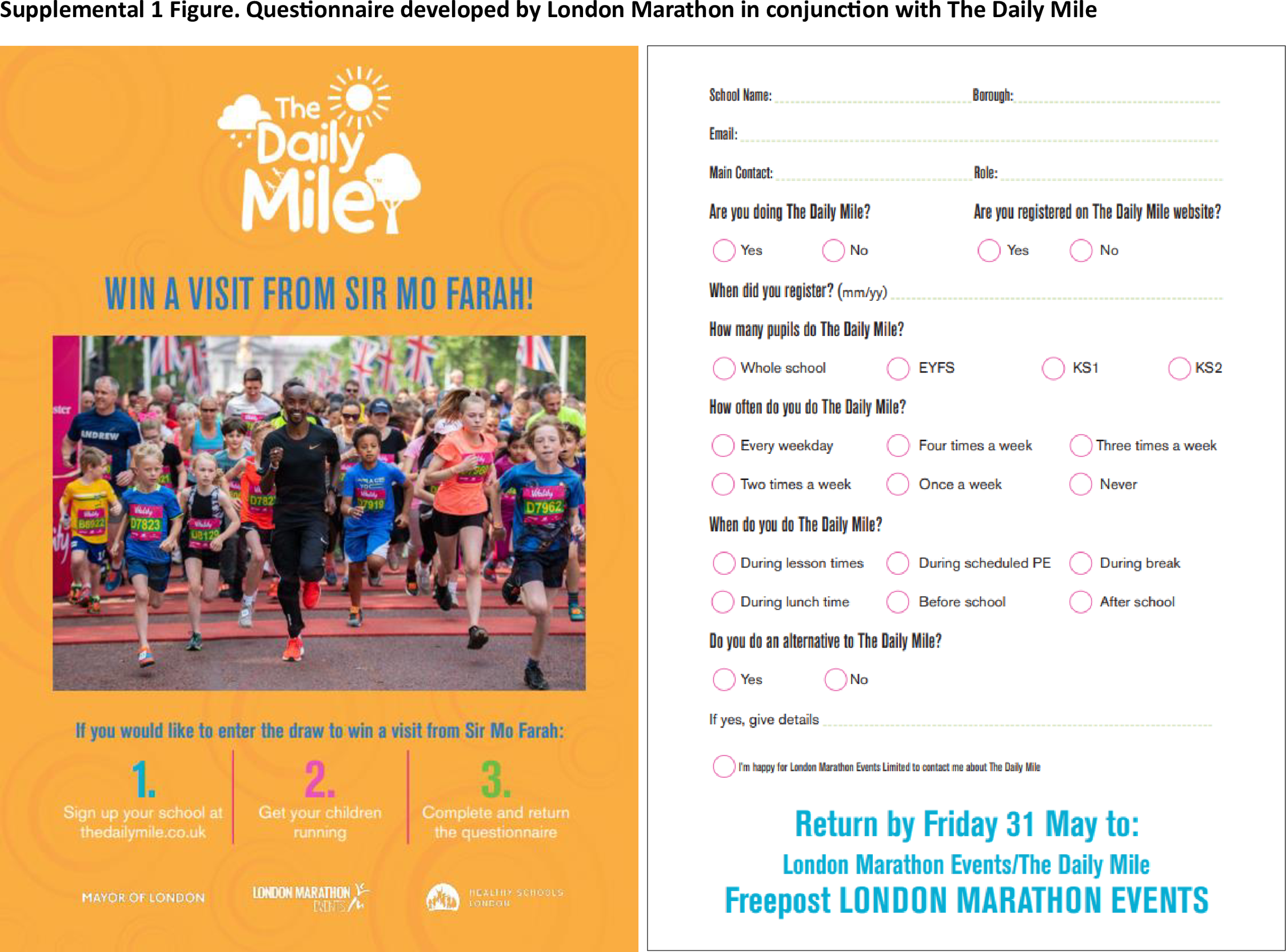

Supplement: S1 Fig — (TIF) [file pone.0288500.s002.tif]

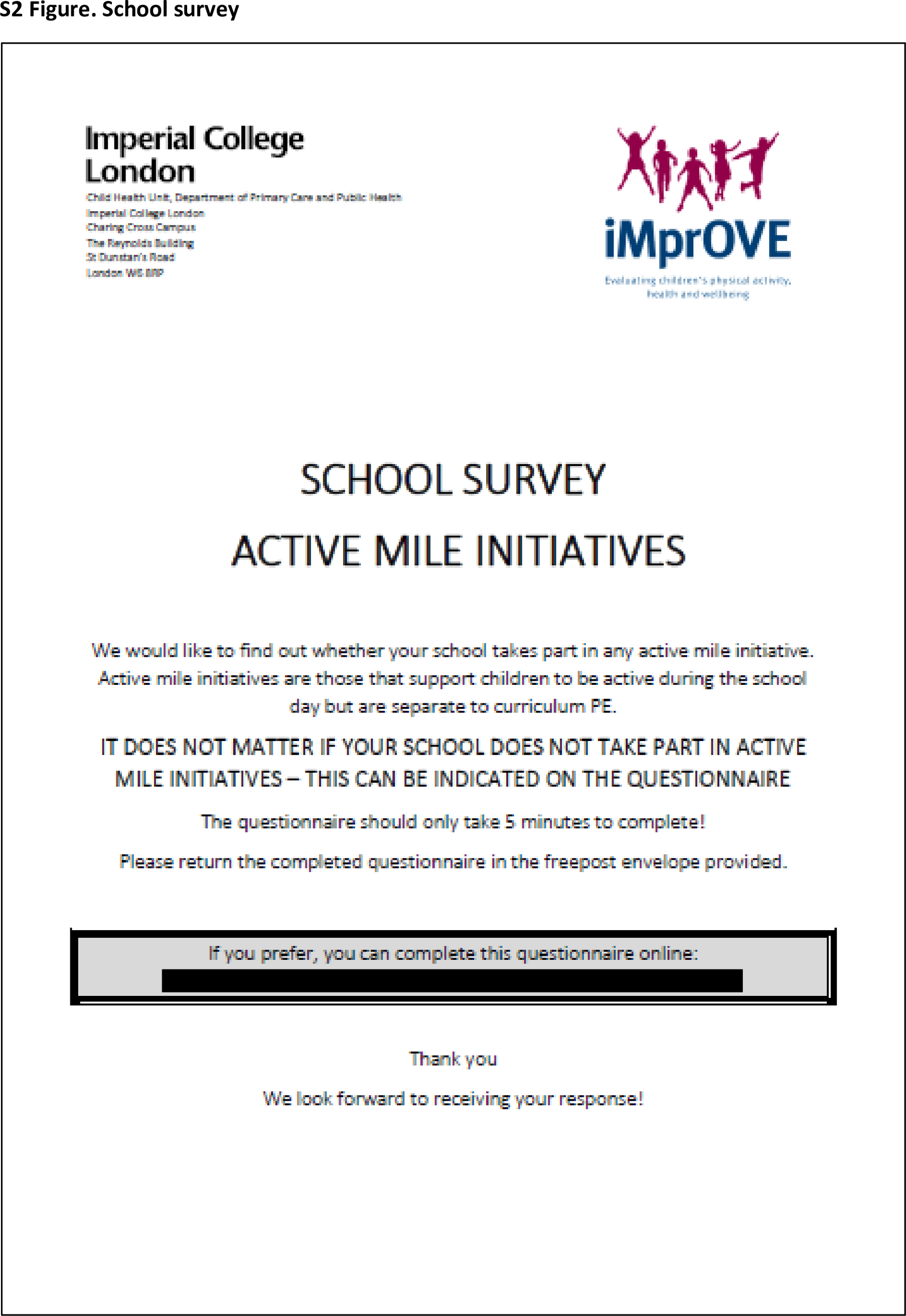

Supplement: S2 Fig — (ZIP) [file pone.0288500.s003.zip › S2 Figure School survey (0).tif]

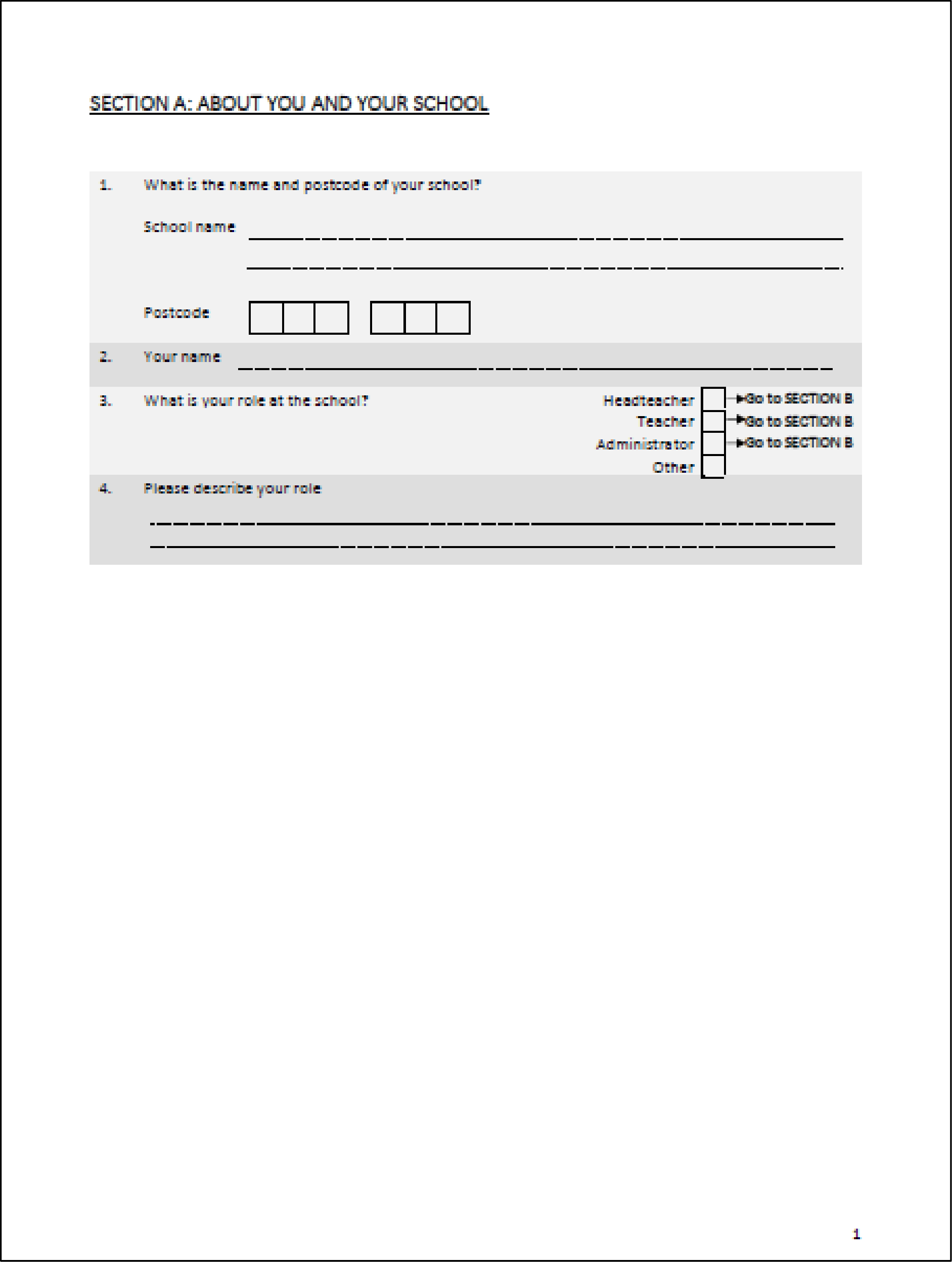

Supplement: S2 Fig — (ZIP) [file pone.0288500.s003.zip › S2 Figure School survey (1).tif]

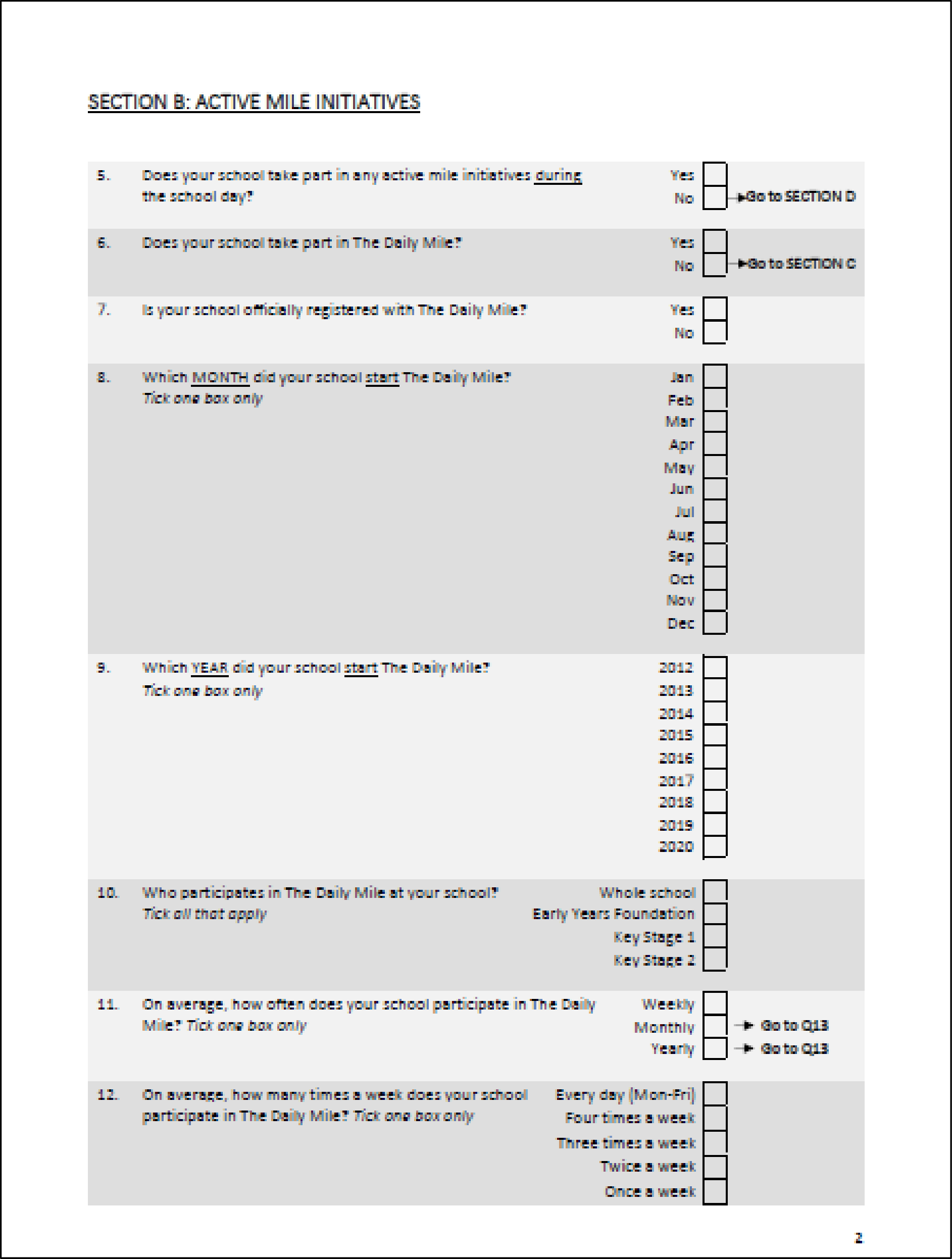

Supplement: S2 Fig — (ZIP) [file pone.0288500.s003.zip › S2 Figure School survey (2).tif]

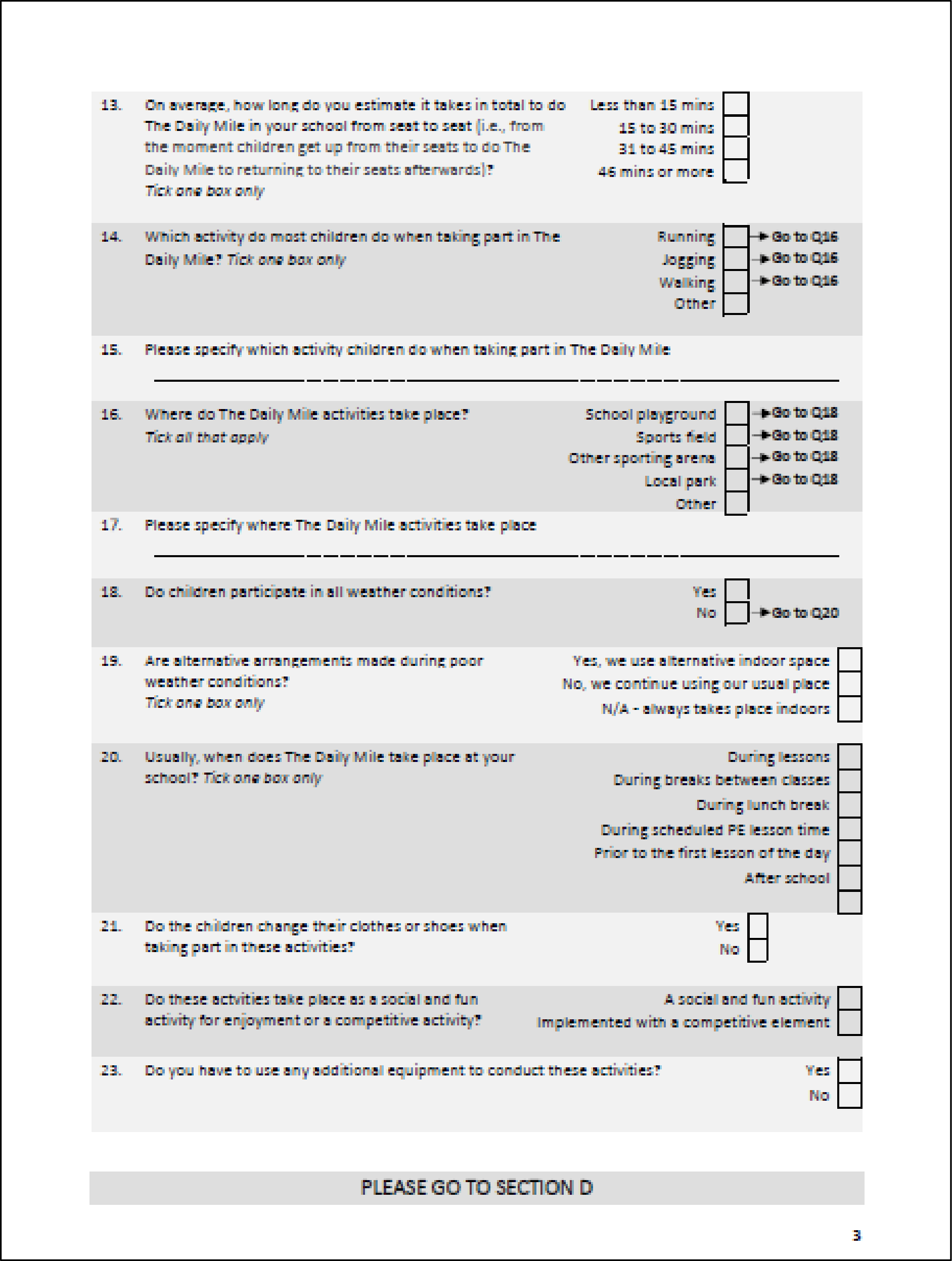

Supplement: S2 Fig — (ZIP) [file pone.0288500.s003.zip › S2 Figure School survey (3).tif]

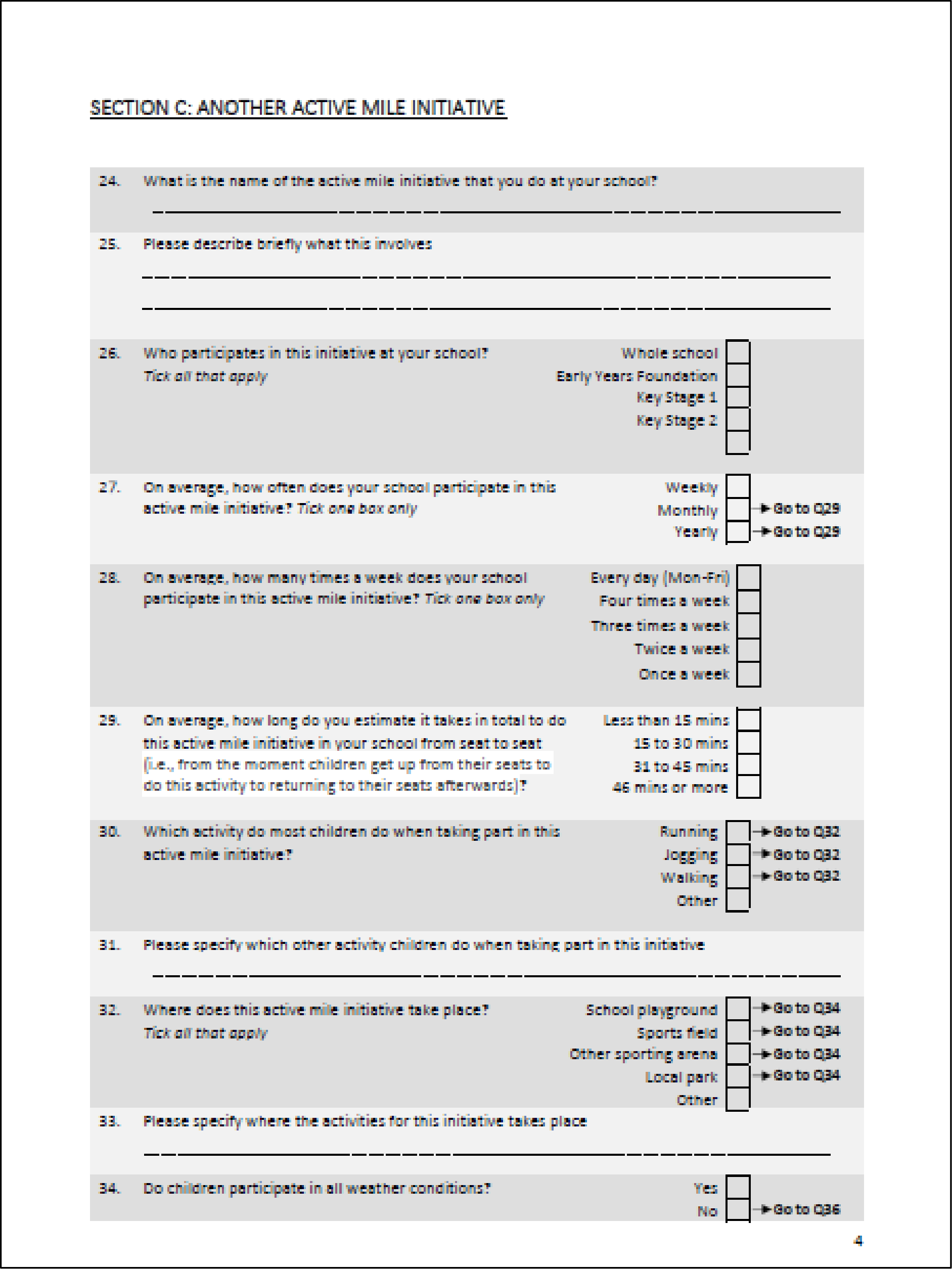

Supplement: S2 Fig — (ZIP) [file pone.0288500.s003.zip › S2 Figure School survey (4).tif]

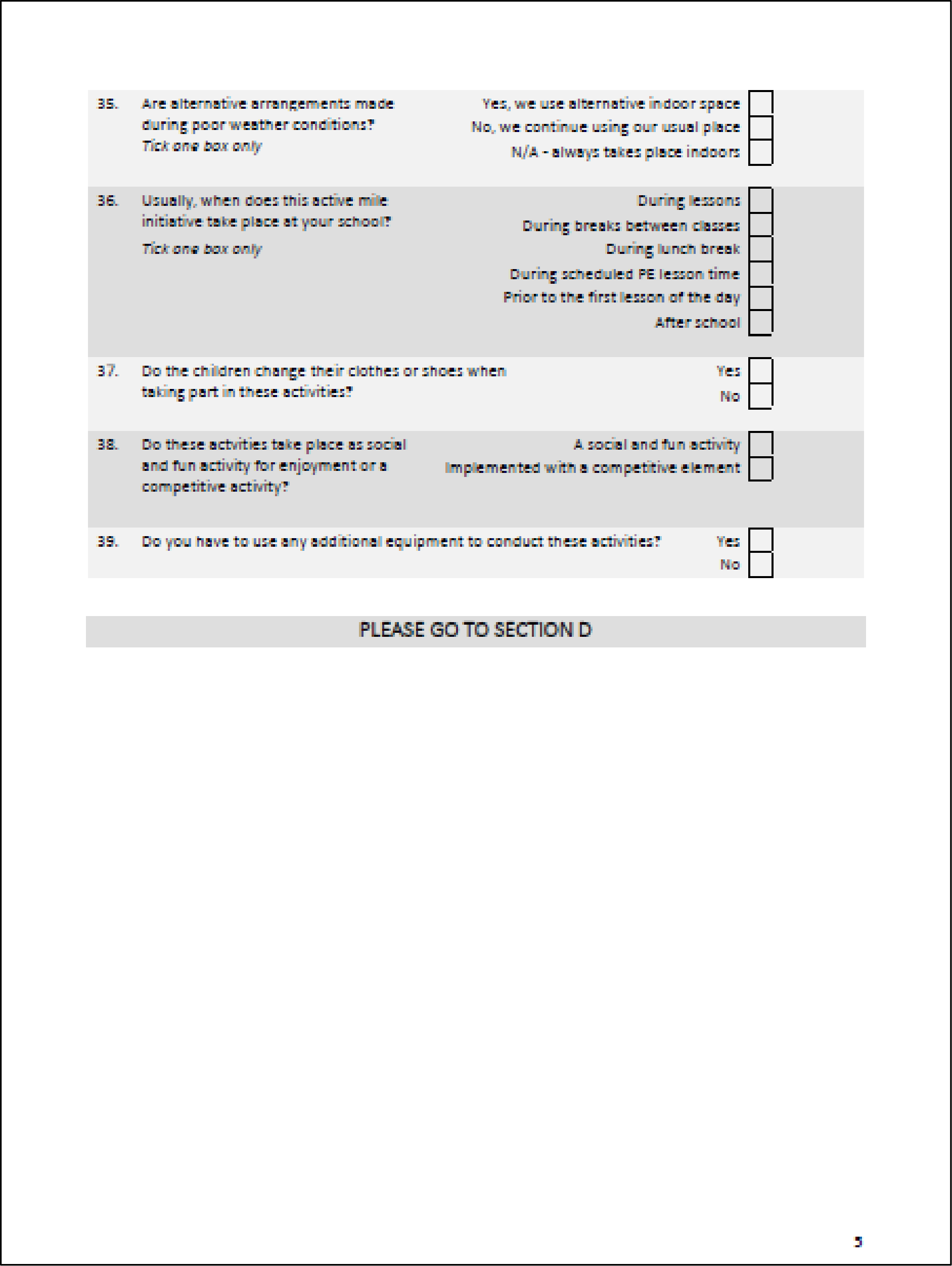

Supplement: S2 Fig — (ZIP) [file pone.0288500.s003.zip › S2 Figure School survey (5).tif]

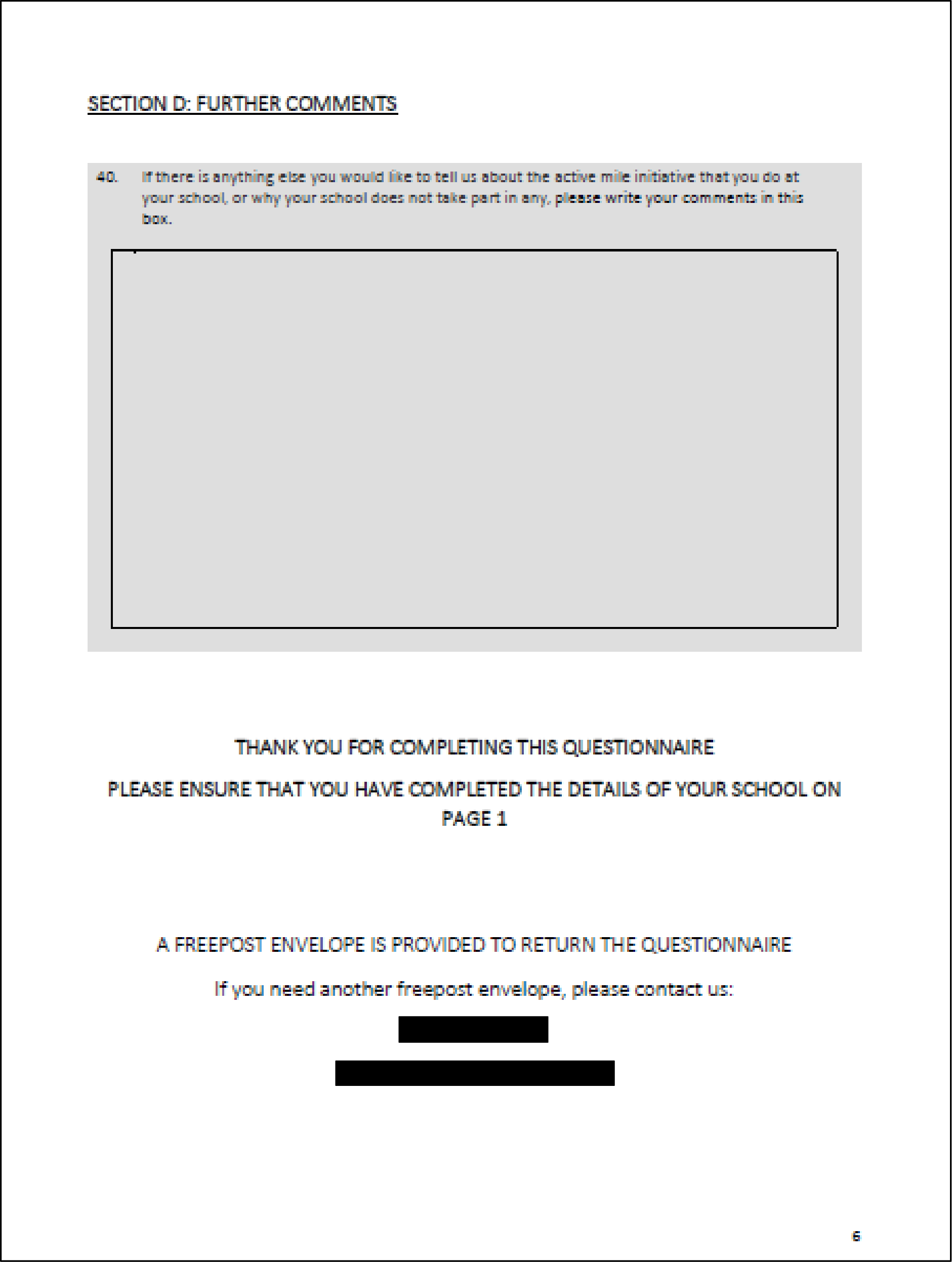

Supplement: S2 Fig — (ZIP) [file pone.0288500.s003.zip › S2 Figure School survey (6).tif]
