# Supplementary material for: Real-world application of a scalable school-based physical activity intervention: A cross-sectional survey of the implementation of The Daily Mile in Greater London primary schools
Source: PLoS One. 2023 Aug 9;18(8):e0288500. doi: 10.1371/journal.pone.0288500 (PMC10411754; doi:10.1371/journal.pone.0288500)
Supplement: S1 Table — (PDF) [file pone.0288500.s004.pdf]

S1 Table. Ofsted ratings[1] and school type[2] descriptions

Ofsted ratings	Description
Outstanding	The school meets all the criteria for a good quality of education securely and consistently, and the quality of education provided is exceptional.
Good	Leaders adopt or construct a curriculum that is ambitious and designed to give all pupils, particularly disadvantaged pupils and pupils with SEND, the knowledge and cultural capital they need to succeed in life. This is either the national curriculum or a curriculum of comparable breadth and ambition.
Requires improvement	The quality of education provided by the school is not good.
Inadequate	The quality of education is likely to be inadequate if for example, the school's curriculum has limited ambition, little or no structure or coherence, and leaders have not appropriately considered content and sequencing.
Rating not available	No data available.
**School type**	
Academy converter schools	Schools which previously received a 'good' or 'outstanding' Ofsted rating and have chosen to convert to academy status.
Academy sponsor led schools	Usually underperforming schools that have chosen to convert to an academy run by sponsors who are responsible for improving the school's performance.
Community schools (also known as local authority-maintained schools)	Schools which are not influenced by businesses or religious groups, and they follow they national curriculum.
Foundation schools	Funded by the government via the local authority and do not charge fees for students.
Free schools	Owned by the government but not run by the local authority.
Voluntary aided	Religious or faith schools.
Voluntary controlled schools	Schools that are maintained by the local authority and often have a religious character.

[1] *Source: UK Government School Inspection Handbook: https://www.gov.uk/government/publications/school-inspection-handbook-eif/school-inspection-handbook#grade-descriptors-for-quality-of-education*
[2]*Source: UK Government. Types of School UK [Available from: https://www.gov.uk/types-of-school]*
