# Supplementary material for: Real-world application of a scalable school-based physical activity intervention: A cross-sectional survey of the implementation of The Daily Mile in Greater London primary schools
Source: PLoS One. 2023 Aug 9;18(8):e0288500. doi: 10.1371/journal.pone.0288500 (PMC10411754; doi:10.1371/journal.pone.0288500)
Supplement: S2 Table — (PDF) [file pone.0288500.s005.pdf]

S2 Table. Grouping survey responses to establish adherence to The Daily Mile's ten core principles

Principle	Response options	Grouping / categories of responses
1 Quick*	1 = less than 15 mins<br>2 = 15 to 30 mins<br>3 = 31 to 45 mins<br>4 = 45 mins or more	• less than 15 mins (response 1)<br>• at least 15 mins (responses 2, 3, & 4)
2 Fun	1 = a social and fun activity<br>2 = competitive element	n/a
3 100%*	1 = Whole school<br>2 = Early Years Foundation<br>3 = Key Stage 1<br>4 = Key Stage 2	• whole school (response 1)<br>• specific year groups (responses 2, 3, & 4)
4 Weather	Yes<br>No	n/a
5 & 6. Route & Risk[a]	1 = school playground<br>2 = sports field<br>3 = other sporting arena<br>4 = local park<br>5 = other	• school playground (response 1)<br>• another area (responses 2, 3, 4, & 5)
7 When to go[b,*]	1 = during lessons<br>2 = during breaks between classes<br>3 = during lunch break<br>4 = during scheduled PE lesson time<br>5 = prior to the first lesson of the day<br>6 = after school	• during lessons (response 1)<br>• other (responses 2, 3, 4, 5 & 6)
	5 = everyday (Mon-Fri)<br>4 = four times a week<br>3 = three times a week<br>2 = twice a week<br>1 = once a week	• once or twice a week (responses 1 & 2)<br>• at least three times a week (responses (3, 4 & 5)
8 Clothes	Yes<br>No	n/a
9 Own pace	1 = running<br>2 = jogging<br>3 = walking<br>4 = other	• running/jogging (responses 1 & 2)<br>• other (responses 3 & 4)
10 Simple	Yes<br>No	n/a

**To establish adherence to the three fundamental principles, responses to Principles 1, 3, and 7 were combined.*

[a]Participants required to indicate 'all that apply'. Those indicating 'school playground' regardless of indicating any additional areas was included in the 'school playground' category. Any other responses or combination of responses (excluding school playground) were grouped together.

[b]Both questions explored separately and then combined to establish adherence to implementation during lessons at least three times a week.
