# Supplementary material for: Real-world application of a scalable school-based physical activity intervention: A cross-sectional survey of the implementation of The Daily Mile in Greater London primary schools
Source: PLoS One. 2023 Aug 9;18(8):e0288500. doi: 10.1371/journal.pone.0288500 (PMC10411754; doi:10.1371/journal.pone.0288500)
Supplement: S3 Table — (PDF) [file pone.0288500.s006.pdf]

S3 Table. Characteristics of schools that did and did not complete the survey

Schools		Completed n=369(%[a])	Not completed n=1348(%[a])	All Schools n=1717(%[a])	p value[b]
Type of school[1]					
	Academy converter	56 (15)	195 (14)	251 (15)	
	Academy sponsor led	16 (4)	95 (7)	111 (6)	
	Community	187 (51)	648 (48)	835 (49)	
	Foundation	15 (4)	26 (2)	41 (2)	
	Free	16 (4)	44 (3)	60 (3)	
	Voluntary aided	78 (21)	336 (25)	414 (24)	
	Voluntary controlled	1 (0.3)	4 (0.3)	5 (0.3)	0.06
Total Pupil Numbers[2]					
	<99	3 (0.8)	10 (0.7)	13 (0.8)	
	100 to 499	292 (79)	1059 (79)	1351 (79)	
	500 to 999	74 (20)	264 (20)	338 (20)	
	>=1000	0	15 (1)	15 (0.9)	0.24
School gender[1]					
	Mixed	1 (0.3)	3 (0.2)	1711 (99)	
	Boys	1 (0.3)	1 (0.1)	4 (0.2)	
	Girls	367 (99)	1344 (99)	2 (0.1)	0.61
Pupil's eligible for SEN support[2]					
	<=99	358 (97)	1272 (94)	1630 (95)	
	100 to 199	11 (3)	74 (5)	85 (5)	
	200 to 299	0	1 (0.1)	1 (0.1)	
	>=300	0	1 (0.1)	1 (0.1)	0.22
Pupil's with English as second language [2]					
	<=99	95 (26)	418 (31)	513 (30)	
	100 to 499	260 (70)	868 (64)	1128 (66)	
	500 to 1000	14 (4)	62 (5)	76 (4)	0.09
Pupil's eligible for free school meals[2]					
	<=99	269 (73)	838 (62)	1107 (64)	
	100 to 199	92 (25)	442 (33)	534 (31)	
	200 to 299	8 (2)	58 (4)	66 (4)	
	300 to 399	0	10 (1)	10 (1)	0.001
OFSTED* rating[1 3]					
	Outstanding	87 (24)	310 (23)	397 (23)	
	Good	253 (67)	914 (68)	1167 (68)	
	Requires improvement	15 (4)	55 (4)	70 (4)	
	Inadequate	0	5 (0.4)	5 (0.3)	
	Rating not available	14 (4)	64 (5)	78 (5)	0.73
Area deprivation IDACI[4] (quintiles)					
	(Most deprived) 1	39 (11)	240 (18)	279 (16)	
	2	93 (25)	350 (26)	443 (26)	
	3	83 (22)	244 (18)	327 (19)	
	4	100 (27)	305 (23)	405 (24)	
	(Least deprived) 5	54 (15)	209 (16)	263 (15)	0.05

[a]May not total 100% due to rounding

[b]Chi-square test

[1]Source: https://www.gov.uk/government/statistics/schools-pupils-and-their-characteristics-january-2021

[2]Source: https://www.gov.uk/government/collections/statistics-performance-tables

[3]Ofsted rating of 'Inadequate' includes schools that are rated 'Serious Weaknesses' or 'Special Measures'

[4]IDACI: Income Deprivation Affecting Children Index

**Ofsted, The Office for Standards in Education, Children's Services and Skills*
