# Supplementary material for: Real-world application of a scalable school-based physical activity intervention: A cross-sectional survey of the implementation of The Daily Mile in Greater London primary schools
Source: PLoS One. 2023 Aug 9;18(8):e0288500. doi: 10.1371/journal.pone.0288500 (PMC10411754; doi:10.1371/journal.pone.0288500)
Supplement: S4 Table — (PDF) [file pone.0288500.s007.pdf]

S4 Table. Optional free-text comments

Principle referenced	Comments
Principle 2: fun and social activity	*"Sometimes we use a theme/fun day (e.g., sport relief, children in need etc) some element of competition".*<br><br>*"We often make it a competition between classes to see which class does the daily mile without stopping the most. We have a trophy and a class prize for the winning class".*<br><br>*"The Daily Mile is completed with a competitive element but is one to do with personal challenge rather than against other children so to help children improve each week".*
Principle 3: 100% (and non-participation)	*"Participation depends on the class teacher: some are really keen to do it with their classes but some classes never do it, as participation is optional".*<br><br>*"Some teachers currently do it with their class but time restrictions and encouraging some staff to start and maintain it consistently are barriers".*<br><br>*"The pressure of getting through the curriculum means the mile gets missed".*<br><br>*"We have a very small playground and hall. There really is no space. Doing it out of school would require large man power and time".*
Principle 7: When to go	*"We started doing the Daily Mile during lesson times, however teachers found that it was taking too long to start and finish our PE teacher started to incorporate it into PE lessons".*<br><br>*"We have implemented the daily mile instead of morning playtime"*<br><br>*"We used to run the mile before school, from 8:30am - 8:45am. We invited parents, carers, grandparents and siblings to join us".*
Principle 8: Change of shoes/clothes	*"Our school uniform has now incorporated black trainers".*<br><br>*"The children come into school on a P.E/sports day in kit so do not require a change of clothes".*

Principle 10: Simple	*"Children lose motivation for the run quickly and end up walking/chatting".*<br><br>*"We do the Daily Mile to Music and end with a dance routine".*<br><br>*"We play music in the summer term to make it more enjoyable".*<br><br>*"…to collect a cube each lap and record how many laps completed. Sometimes use equipment e.g., throwing / bouncing a ball around".*<br><br>*"The children became bored of just running so we have limited the 'mile' running but tried to increase children's activity levels in the classroom and during playtimes with different offerings of equipment, challenges and play leaders".*
Positive experiences of participating in The Daily Mile	*"The Daily Mile is a wonderful initiative which all of the staff and children enjoy. It acts as a refresh or restart enabling the children to focus while also enabling them to socialize and stay fit and healthy".*<br><br>*"The children really enjoy the daily mile whether they walk, jog or run".*<br><br>*"We complete the daily mile as it makes the children healthier and fitter and improves concentration for when they are back in the classroom".*<br><br>*"Children enjoy the brain break".*
